# The relapsed acute lymphoblastic leukemia network (ReALLNet): a multidisciplinary project from the spanish society of pediatric hematology and oncology (SEHOP)

**DOI:** 10.3389/fped.2023.1269560

**Published:** 2023-09-20

**Authors:** Pablo Velasco, Francisco Bautista, Alba Rubio, Yurena Aguilar, Susana Rives, Jose L. Dapena, Antonio Pérez, Manuel Ramirez, Cristina Saiz-Ladera, Elisa Izquierdo, Adela Escudero, Mireia Camós, Nerea Vega-García, Margarita Ortega, Gloria Hidalgo-Gómez, Carlos Palacio, Pablo Menéndez, Clara Bueno, Joan Montero, Paola A. Romecín, Santiago Zazo, Federico Alvarez, Juan Parras, Carmen Ortega-Sabater, Salvador Chulián, María Rosa, Davide Cirillo, Elena García, Jorge García, Albert Manzano-Muñoz, Alfredo Minguela, Jose L. Fuster

**Affiliations:** ^1^ Pediatric Oncology and Hematology Department, Vall d’Hebron Barcelona Hospital, Campus, Barcelona, Spain; ^2^ Trial and Data Centrum, Prinses Maxima Centrum, Princess Máxima Center for Pediatric Oncology, Utrecht, Netherlands; ^3^ Pediatric Oncology and Hematology Department, Hospital Infantil Universitario Niño Jesús, Madrid, Spain; ^4^ Pediatric Oncology and Hematology Department, Hospital Miguel Servet Hospital, Zaragoza, Spain; ^5^ Leukemia and Lymphoma Unit, Pediatric Cancer Center Barcelona (PCCB), Hospital Sant Joan de Déu de Barcelona, Barcelona, Spain; ^6^ Pediatric Cancer Center Barcelona (PCCB), Institut de Recerca Sant Joan de Déu, Leukemia and Pediatric Hematology Disorders, Developmental Tumors Biology Group, Barcelona, Spain; ^7^ Translational Research in Pediatric Oncology, Hematopoietic Transplantation and Cell Therapy Group, Hospital La Paz Institute for Health Research (IdiPAZ), La Paz University Hospital, Madrid, Spain; ^8^ Pediatric Hemato-Oncology Department, La Paz University Hospital, Madrid, Spain; ^9^ Pediatric Department, Universidad Autonoma de Madrid, Madrid, Spain; ^10^ Hematology and Oncology Laboratory, Fundación Para La Investigación Biomédica Hospital Infantil Universitario Niño Jesús, Madrid, Spain; ^11^ Department of Genetics, Institute of Medical and Molecular Genetics (INGEMM), La Paz University Hospital, Madrid, Spain; ^12^ Centro de Investigación Biomédica en Red de Enfermedades Raras (CIBERER), Instituto de Salud Carlos III, Madrid, Spain; ^13^ Hematology Laboratory, Hospital Sant Joan de Déu Barcelona, Barcelona, Spain; ^14^ Hematology Service, Vall d’Hebron Barcelona Hospital, Campus, Barcelona, Spain; ^15^ Vall d’Hebron Institute of Oncology (VHIO), Vall d’Hebron Barcelona Hospital Campus, Barcelona, Spain; ^16^ Josep Carreras Leukemia Reserach Institute, Developmental Leukemia and Immunotherapy group, Barcelona, Spain; ^17^ Red Española de Terapias Avanzadas (TERAV)-Instituto de Salud Carlos III (ISCIII) (RICORS, RD21/0017/0029), Madrid, Spain; ^18^ CIBER-ONC, ISCIII, Barcelona, Spain; ^19^ Institució Catalana de Recerca I Estudis Avançats (ICREA), Barcelona, Spain; ^20^ Department of Biomedicine, School of Medicine, University of Barcelona, Barcelona, Spain; ^21^ Networking Biomedical Research Center in Bioengineering, Biomaterials and Nanomedicine (CIBER-BBN), Madrid, Spain; ^22^ Department of Biomedical Sciences, Faculty of Medicine and Health Sciences, Universitat de Barcelona, Barcelona, Spain; ^23^ Information Processing and Telecommunications Center, Universidad Politécnica de Madrid, Madrid, Spain; ^24^ Mathematical Oncology Laboratory (MOLAB), University of Castilla-La Mancha, Ciudad Real, Spain; ^25^ Department of Mathematics, Universidad de Cádiz, Cádiz, Spain; ^26^ Biomedical Research and Innovation Institute of Cádiz (INiBICA), Hospital Universitario Puerta del Mar, Cádiz, Spain; ^27^ Barcelona Supercomputing Center (BSC), Barcelona, Spain; ^28^ Nanobioengineering Group, Institute for Bioengineering of Catalonia (IBEC), Barcelona Institute of Science and Technology (BIST), Barcelona, Spain; ^29^ Immunology Department, Hospital Clínico Universitario Virgen de la Arrixaca, Murcia, Spain; ^30^ Instituto Murciano de Investigación Biosanitaria (IMIB), Murcia, Spain; ^31^ Paediatric Oncohematology Department. Hospital Clínico Universitario Virgen de la Arrixaca, Murcia, Spain

**Keywords:** relapsed acute lymphoblastic leukemia, precision medicine, cancer registry, artificial intelligence, functional assay

## Abstract

Acute lymphoblastic leukemia (ALL) is the most common pediatric cancer, with survival rates exceeding 85%. However, 15% of patients will relapse; consequently, their survival rates decrease to below 50%. Therefore, several research and innovation studies are focusing on pediatric relapsed or refractory ALL (R/R ALL). Driven by this context and following the European strategic plan to implement precision medicine equitably, the Relapsed ALL Network (ReALLNet) was launched under the umbrella of SEHOP in 2021, aiming to connect bedside patient care with expert groups in R/R ALL in an interdisciplinary and multicentric network. To achieve this objective, a board consisting of experts in diagnosis, management, preclinical research, and clinical trials has been established. The requirements of treatment centers have been evaluated, and the available oncogenomic and functional study resources have been assessed and organized. A shipping platform has been developed to process samples requiring study derivation, and an integrated diagnostic committee has been established to report results. These biological data, as well as patient outcomes, are collected in a national registry. Additionally, samples from all patients are stored in a biobank. This comprehensive repository of data and samples is expected to foster an environment where preclinical researchers and data scientists can seek to meet the complex needs of this challenging population. This proof of concept aims to demonstrate that a network-based organization, such as that embodied by ReALLNet, provides the ideal niche for the equitable and efficient implementation of “what's next” in the management of children with R/R ALL.

## 1. Introduction

Acute lymphoblastic leukemia (ALL) is the most common childhood cancer. Around 300 new cases are diagnosed each year in Spain, representing one-quarter of all pediatric malignancies. In Spain, 85% of patients will survive in the long-term (1). However, 15% will experience a first relapse or will be refractory to first line chemotherapy (R/R ALL) and, for those, the overall survival drops below 50% and 30% respectively (2, 3). As a consequence, ALL remains one of the main causes of cancer death among children (4).

The International Berlin-Frankfurt-Münster (I-BFM) Study Group and the Spanish Society of Pediatric Hematology and Oncology (SEHOP) have developed treatment protocols for pediatric R/R ALL to improve these statistics. In 2015, the LAL/SEHOP-PETHEMA-2015 guidelines were established by the ALL-working group of SEHOP. This guideline is based on the standard protocols from the IntReALL 2010 standard and high-risk trials (NCT01802814, NCT03590171) and it aimed to harmonize the treatment of children with ALL at first relapse in Spain. The data gathered from the aforementioned protocols and guideline have demonstrated that the leukemia genomics, time elapsed from first diagnosis to relapse, location of relapse, and response after re-induction in terms of minimal residual disease (MRD), are critical in defining risk groups that may require more intensive or alternative treatments, such as immunotherapy or targeted therapies (4–6). Based on these findings, upcoming clinical trials in this population will rely on the use of advanced genetic studies and standardized MRD monitoring for risk stratification; however, not all centers in Spain have access to the necessary technology to perform these techniques.

The LAL/SEHOP/PETHEMA-2015 guideline provided the first registry of clinical data from pediatric patients after first relapse of ALL in Spain; nevertheless, a more comprehensive registry collecting accurate biological and clinical data that covers the entire population is needed to have a broader view of the situation in our country that can be used as a baseline for future comparison. Moreover, it is necessary to create a nationwide biobank where tumor material can be stored for further research that can be coupled with other clinical and biological data. This is a fundamental piece to promote research in this field in Spain, and to overcome the historical limitations of the preclinical researchers to have access to samples necessary to perform high-quality research.

Recently, the European Society for Pediatric Oncology published the 2021–2026 European Strategic Cancer Plan for Children and Adolescents, which focused on the mission to “cure more and cure better” (7). To this end, they developed seven research objectives centered on equity, precision medicine, understanding the biology and causes of cancer, improving quality of life, and leveraging big data and artificial intelligence.

In line with these principles, at a European level, academic societies like the Innovative Therapies for Children with Cancer (ITCC) consortium (8) and, in Spain, the International Multicenter Clinical Trial-SEHOP (ECLIM-SEHOP) platform (9), work to enable access to new therapies through clinical trials. Moreover, experts in R/R ALL from both I-BFM and ITCC are currently working on establishing committees designed to discuss complex cases referred from national boards, like the FEDRRAL tumor board from IBFM resistant disease group, or the international leukemia target board (iLTB), where children with relapsed/refractory hematological malignancies, are discussed (NCT05270096).

All this innovation in the diagnosis and treatment of R/R ALL has increased the complexity in managing these patients, which represents a significant challenge for centers to implement and interpret the most current diagnostic methods and to access clinical trials of new drugs.

The Relapsed Acute Lymphoblastic Leukemia Network (ReALLNet) is a project established under the umbrella of SEHOP in March 2021. The goal of ReALLNet is to bridge the gap between bedside patient care and the clinical and research expert field. This will bring innovation quickly and comprehensively to all centers across Spain in an equitable manner. Simultaneously, it seeks to influence the promotion and organization of research studies, ensuring they are driven by real healthcare needs. The methods to achieve this include: (1) establishing a network of laboratories for conducting biological studies and advanced research, (2) consolidating a network of hospitals able of providing access to clinical trials, and (3) establishing a shared data resource and biobank for R/R ALL samples.

In the following subsections, we present the structure upon which ReALLNet has been built to address these objectives, as well as the preliminary results of the network and forthcoming steps.

## 2. Relapse and refractory ALL national registry

The cancer registry offers several benefits. It aids in identifying risk factors, informs clinical practice and treatment decisions, evaluates the effectiveness and outcomes of treatments, facilitates research and collaboration, monitors trends and disparities, and serves as a basis for guidelines and public health interventions (10).

To catalog and record all the information generated, a national registry for pediatric R/R ALL has been created. This registry systematically collects clinical and biological data from patients in a coded format. This was created using the RedCap system (11), hosted on the servers of Vall d'Hebron Research Institute (VHIR).

Upon the signing of informed consent by the patient and their family, the treating physician can access the registry, where the patient will be identified by a unique code. Variables will be entered following the patient's course with R/R ALL, including first diagnosis, first and subsequent relapses or refractoriness. The user will be allowed to access their data and graphs, which enhances the registrant's experience and facilitates its use as their own local registry. In the following stages, it is planned to incorporate Patient-Reported Outcome Measures (PROMs) and Patient-Reported Experience Measures (PREMs) into the registry to generate additional outcome information to better respond to patient's preferences.

## 3. ReALL board

As an initial step, the board was constituted by experts in R/R ALL from clinical, diagnostic, research, and mathematical and computational fields. In October 2021, the ReALLNet platform was launched within the secured environment of the SEHOP website (www.sehop.org) for professionals. This platform offers various resources such as a discussion forum where physicians from any Spanish center can submit inqueries about specific cases and participate in discussions within 48 h of consultation. The data of this consultations are not included in the registry.

Twenty-four patients from 15 regions were discussed between October 2021 and July 2023 (Table 1). Patients' ages ranged from 8 months to 16 years, with most of them having a poor prognosis R/R ALL, 6 patients had high-risk first relapse of ALL (early and very early), 6 had a second or later relapse, and 3 had refractory disease. Twenty-three treating physicians followed the recommendations of the ReALLBoard. Immunotherapy was administered in seven patients, and targeted therapy based on functional assays was given in two patients. The board discussions resulted in a total of 15 referrals, involving both patients and samples, to the identified centers of expertise within ReALLNet. Out of these referrals, 2 patients were directed to participate in a clinical trial, 3 patients were referred for CAR-T therapy, and 3 patients were referred for hematopoietic stem transplantation (HSCT). In terms of samples, 5 samples were referred for functional assays by drug response profiling (DRP) and dynamic BH3 profiling (DBP), while 2 samples were referred for next-generation sequencing (NGS) analysis. At data cut-off (July 15th, 2023), 18 patients remain alive and in complete remission (CR), 1 is alive with disease after subsequent relapse, and 5 patients have died, 3 of them due to disease progression and 2 due to treatment-related toxicity.

**Table 1:** Description of cases discussed in the ReALLBoard, recommendations, adherence to them, referrals of patients or samples, and outcome.

Case	Age (y)	ALL lineage	Date of ReALLBoard	Cytogenetics	Relapse or refractory criteria	Recommendation	Adh	Referral to another center	Current status
1	4	B-ALL	Nov 2021	Hyperdiploidy. CRLF2 rearrangement	Very early isolated bone marrow first relapse	LAL/SEHOP-PETHEMA 2015 HR and blinatumomab	Yes	No	Alive, CR
2	10	T-ALL	Dec 2021	NOS	Isolated bone marrow fourth relapse	Venetoclax + bortezomib after DRP/DBP	Yes	Sample, for DRP/DBP	Death of disease
3	11	B-ALL	Jan 2022	ETV6::RUNX1	Isolated bone marrow second relapse	CAR-T	Yes	Patient, for CAR-T	Alive, CR
4	0.6	B-ALL	Jan 2022	KMT2A::AFF1	Refractory combined first relapse	CAR-T and DRP/DBP	Yes	Sample, for DRP/DBP	Death of disease
5	16	B-ALL	Jan 2022	ETV6::RUNX1	Very early isolated bone marrow first relapse	LAL/SEHOP-PETHEMA 2015 HR and blinatumomab	Yes	No	Alive, CR
6	5	B-ALL	Feb 2022	NOS	Early isolated bone marrow first relapse	LAL/SEHOP-PETHEMA 2015 HR	Yes	No	Alive, CR
7	13	B-ALL	Mar 2022	Hyperdiploidy. TP53 mut	Refractory isolated bone marrow first relapse	CAR-T	Yes	Patient, for CAR-T	Alive, CR
8	9	T-ALL	May 2022	ETP	Early isolated bone marrow first relapse, after HSCT	Venetoclax ater DRP/DBP and HSCT	Yes	Sample, for DRP/DBP	Alive, CR
9	4	B-ALL	May 2022	ETV6::RUNX1	Refractory isolated bone marrow first relapse	Blinatumomab, Inotuzumab and HSCT	Yes	Patient, for HSCT	Alive, CR
10	16	T-ALL	Aug 2022	NOS	MRD persistence during first line treatment	Nelarabine and HSCT	Yes	Patient, for HSCT	Alive, CR
11	8	T-ALL	Aug 2022	NOS	Early extramedullary (ocular) second relapse after HSCT	Nelarabine, radiotherapy, IT	Yes	No	Death of disease
12	11	MPAL	Sep 2022	ZEB::BCL11B	Late isolated bone marrow first relapse, after HSCT	TVTC	No	No	Treatment related death
13	11	T-ALL	Oct 2022	CDKN2A del	Re-emergence, MRD 1%	CVC and HSCT	Yes	Sample, for NGS	Treatment related death
14	9	B-ALL	Nov 2022	FLT3 mut	Re-emergence during midostaurin maintenance	Follow up	Yes	Sample, for DRP/DBP	Alive with disease
15	10	B-ALL	Mar 2022	BCR::ABL1	Combined bone marrow second relapse	Ponatinib (CT) +IT	Yes	Patient, for CT	Alive, CR
16	5	B-ALL	Mar 2023	NOS	Late isolated bone marrow first relapse	LAL/SEHOP-PETHEMA 2015 SR	Yes	No	Alive, CR
17	8	B-ALL	Mar 2023	NOS	Late combined bone marrow first relapse	LAL/SEHOP-PETHEMA 2015 SR	Yes	No	Alive, CR
18	3	B-ALL	Mar 2023	NOS	Early isolated medullary second relapse	CAR-T	Yes	Patient, for CAR-T	Alive, CR
19	14	B-ALL	Apr 2023	KRAS mut	Poor response to induction of late isolated bone marrow first relapse	LAL/SEHOP-PETHEMA 2015 HR and blinatumomab	Yes	No	Alive, CR
20	9	B-ALL	Apr 2023	Hyperdiploidy. JAK2 mut, IKZF1 del, PAX5mut	Late combined bone marrow first relapse	LAL/SEHOP-PETHEMA 2015 SR	Yes	No	Alive, CR
21	10	T-ALL	Apr 2023	NOS	Very early isolated bone marrow first relapse	CT	Yes	Patient, for CT	Alive, CR
22	12	T-ALL	May 2023	NOS	Early isolated bone marrow first relapse	LAL/SEHOP-PETHEMA 2015 HR	Yes	Sample, for FC	Alive, CR
23	4	B-ALL	Jul 2023	TCF3::PBX1	Early isolated bone marrow second relapse after CAR-T and HSCT	NGS and DRP/DBP	Yes	Sample, for NGS and DRP/DBP	Alive, CR
24	16	B-ALL	Jul 2023	CRLF2 overexpressed	Late isolated bone marrow first relapse	LAL/SEHOP-PETHEMA 2015 SR	Yes	No	Alive, CR

Adh, adherence; CR, complete response. CT, clinical trial; CVC, clofarabine, etoposide, and cyclophosphamide; DBP, B H3 profiling; DRP, drug response profiling; ETP, early T-cell precursor leukemia; FC, flow cytometry; HSCT, hematopoetic stem cell transplantation; IT, intrathecal therapy; NGS, next generation sequency; NOS, not otherwise specified; MPAL, mixed phenotype acute leukaemia; MRD, minimal residual disease; TVTC, topotecan, vinorelbine, thiotepa, and clofarabine.

## 4. Platform for shipping, analysis, and biobanking of samples

**Figure 1:**
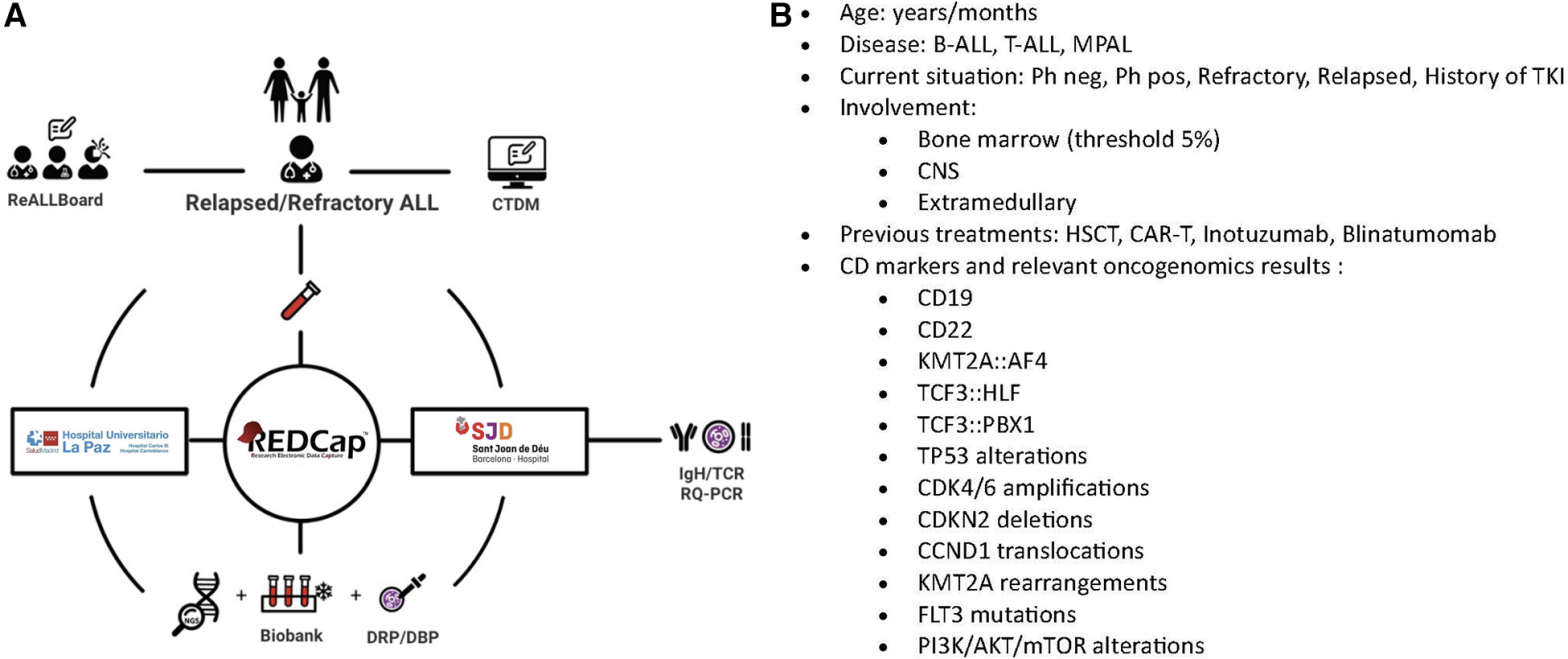
(**A**) Logistical representation of the ReALLNet R/R ALL network on the SEHOP website for professionals. (**B**) Illustrates the algorithm of questions followed by the Clinical Trial Decision Program to match patients with the most suitable trials. Schematic representation of the resources provided by the platform, each represented by an icon. Selecting each icon allows access to the respective resource. ReALLBoard: Access for consultations to the expert committee. CTDM (Clinical Trial Decision Maker): A decision-making program providing the best clinical trials tailored to individual patient needs. Test Tube Icon: Represents the necessary documents for sending samples for diagnosis and preservation (Biobank). This process takes place at the ReALLNet diagnostics hubs, La Paz University Hospital (Madrid) and San Joan de Déu Hospital (Barcelona). RedCAp: A pseudonymized registry of clinical and diagnostic variables for pediatric patients with R/R ALL. IgH/TCR RQ-PCR: Real-time PCR-based quantification of IG/TR rearrangements. NGS: Next Generation Sequencing. Drug Response Profiling: Functional assay at the Institut de Recerca de Josep Carreras (Pablo Menéndez Lab). Dynamic BH3 Profiling: Functional assay at the University of Barcelona (Joan Montero Lab).

To facilitate the referral of samples, ReALLNet has created a sample shipping protocol that ensures the transfer of biological material (i.e., bone marrow relapse, peripheral blood, and germ tissue) within 24 h across the entire Spanish territory. This protocol prioritizes the fresh delivery of samples unless a reception within 48 h of extraction cannot be guaranteed, in which case frozen samples are prioritized. Once the samples arrive at the diagnostic hubs (Hospital Sant Joan de Déu in Barcelona, Hospital Universitario La Paz in Madrid), they are processed for storage in the biobank, oncogenomic tests (Section 5) and functional assays (Section 6). To expedite decision-making and sample referral, a comprehensive logistical process has been devised and graphically represented. This scheme provides detailed instructions on how to access all these resources and is available to all treating physicians via the private SEHOP website (Figure 1A).

## 5. Oncogenomic testing

Several genetic alterations have been demonstrated to predict unfavorable outcomes and, consequently, can benefit from innovative strategic treatments such as immunotherapy or targeted therapies (5, 12, 13). Next generation sequencing (NGS) is currently applied in some centers to identify these alterations. However, numerous centers lack the availability or funding for molecular profile by NGS in their region. In these situations, ReALLNet's highly experienced oncogenomics team processes the submitted samples in its integrated diagnostic units. To this end, we evaluated the commercial and custom NGS panels available in Spanish territory, achieved consensus on the minimal genes to be analyzed in the context of R/R ALL, and created a common report sheet.

An integrative diagnostic committee has been established, appointed to analyze, discuss and report oncogenomic and functional results coming from the network's diagnostic hubs or individual centers.

Similar molecular platforms have been established in other countries, like the LEAP consortium project (14) in the US and INFORM (15) in Germany, showing that between 8% and 12% of patients with a relapsed malignancy can benefit from a targeted treatment based on oncogenomic studies and guided by a multidisciplinary national board.

In subsequent steps, we aim to incorporate additional techniques for a more comprehensive analysis in the network's hubs. In ascending order of complexity, these techniques will include whole-exome sequencing (WES), transcriptome sequencing (RNA-seq), and whole-genome sequencing (WGS).

In addition to identifying tumor DNA variants in patients with R/R ALL, we highlight the importance of complementing genetic studies with the analysis of germline DNA from these patients. In compliance with the principles of the Helsinki Declaration, informed consent is obtained from all participants, ensuring ethical conduct throughout the research process. The identification of germline alterations using, for example, cultured fibroblasts, peripheral blood, or saliva in remission could help to recognize cancer predisposition syndromes (16–18) that increase the risk of leukemia development, often underdiagnosed but critical for the correct management of patients and their families. The germline analysis also allows the identification of genetic polymorphisms that can significantly influence the uptake, metabolism, and elimination of currently used drugs, providing additional helpful information during the treatment of pediatric patients (19, 20). Germinal genetic results analyzed will be reported from the genetic counseling units of the diagnostic centers, in case relevant findings are discovered.

The monitoring of MRD has proven to be a predictive factor in R/R ALL and has been utilized to intensify treatment in ALL relapse protocols (5, 21). Clone-specific MRD assessment has emerged as highly sensitive and has been selected for use in several upcoming clinical trials in R/R ALL. Therefore, ReALLNet seeks to assist centers that have not yet implemented the real-time PCR-based quantification of IG/TR rearrangements for MRD assessment by providing this service through its diagnostic hubs.

## 6. Functional assays

Innovative techniques are employed to evaluate specific leukemia drug sensitivity through *ex vivo* functional studies, such as DRP or DBP. Carrying out *in vitro* cytotoxicity tests on the neoplastic cells could help the search for a more personalized therapeutic option. These studies are performed in real time, yielding results within a short time frame. With this approach we aim to validate its potential to assist clinicians in personalizing therapy, stratifying patients, and identifying more effective and less toxic treatments for R/R ALL. The objective of ReALLNet is to validate these studies through this research. In the meantime, the results are reported as experimental findings awaiting validation.

### 6.1. Dynamic BH3 profiling

Pediatric leukemia often presents resistance to apoptosis (many genetic alterations in ALL lead to an increase in anti-apoptotic proteins of the BCL-2 family). In this project, we use new functional assays to create a drug response profile, including the DBP, which can predict which treatments will be most effective in eliminating a specific tumor. DBP has already been used to evaluate therapies in different types of cancer (22) and has been successfully tested in ALL (23–26). In addition, we can execute this assay to identify anti-apoptotic adaptations and use that information to design new therapeutic strategies for ALL, preventing it from becoming resistant and restoring its sensitivity to cell death.

### 6.2. Drug response profiling

Furthermore, an additional DRP method analyzes cell death engagement on cancer cells co-cultured with mesenchymal stem cells (MSC) isolated from bone marrow (BM). It is well-known that BM stroma confers chemoresistance to leukemic cells in a variety of hematological malignancies (27, 28). In order to mimic BM microenviroment, the assays are carried out by co-cultivating leukemic cells with BM- MSCs. Increasing doses of the drugs to be tested will be added to this co-culture, alone or in combination with the standard chemotherapy according to the type of leukemia. Drugs, which are interrogated, are chosen based on FDA and/or EMA approved medications that specifically target hematologic malignancies such us ruxolitinib, venetoclax, fludarabine, quizartimib, bortezomib, among others. Blasts-MSC co-cultures are incubated for 36 h with each drug combination, the degree of apoptosis or cell viability is detected by flow cytometry.

## 7. Data and computational science

### 7.1. Data science

Early identification of therapeutic scheme failures is crucial to prevent toxicity and initiate alternative treatments promptly. To achieve this, we propose a research work package focused on biomathematical modeling. Our approach involves conducting in silico analysis of patient characteristics associated with the actual disease, enabling the identification of key biomarkers for predictive purposes. The term “*in silico*” refers to the use of computer simulations and data analysis to develop or evaluate medicinal products or medical interventions (29).

Leveraging innovative techniques from machine learning (ML) and artificial intelligence (AI), data science methods could contribute an additional layer to patient classification. These techniques can perform feature extraction, gleaning relevant insights from raw data, and potentially leading to more sophisticated patient stratification and improved healthcare outcomes. By extracting clinical, genomic, and immunophenotypic information, we can label each patient based on their response or disease subtype, creating a labeled dataset that can be analyzed using supervised algorithms. To address potential variations in the number of patient sub cohorts and select the optimal parameters for each model, we will employ k-fold cross-validation techniques. Some methodologies have already explored the use of flow cytometry data, leading to the emergence of computational flow cytometry as a growing field (30). Complementary to flow cytometry, genomic data from these patients will also be available at the time of relapse. They have proven useful to characterize mutability hotspots (31) and to characterize the relative functional impact of driver and passenger mutations (32). Some genomic features, such as copy number alterations (CNA), tumor mutational burden (TMB) or neoantigen load, will provide us with general information on the heterogeneity and disease progression status of these patients. As they are also quantitative measures, they could easily be integrated into supervised algorithms and predictive models, together with flow cytometry data.

However, certain limitations persist, including challenges in translating these studies to clinical settings in an interpretable manner for clinicians. The inclusion of a biomathematical branch in this project will provide user-friendly tools for implementation in hospitals. Constructing predictive algorithms will also require addressing several open problems, including data normalization, methods for imputing missing data, model comparison, feature selection, and interpretation. These challenges are pertinent to any medical dataset and must be adequately resolved to develop robust and clinically relevant predictive models.

### 7.2. Clinical trial decision maker

In order to keep treating physicians up-to-date with the open clinical trials in Spain and to facilitate the discussion of clinical cases within the ReALLBoard, we have developed a decision-support software program, addressed to the attending physicians, known as the Clinical Trial Decision Maker (CTDM). Based on a series of mutually exclusive questions related to the clinical and biological characteristics of the disease, the algorithm provides a list of potential clinical trials for which the patient may be eligible and that are available in any of the SEHOP hospitals (Figure 1B). This method is anticipated to favor the access to patients to clinical trials, thereby broadening the array of opportunities accessible to patients.

## 8. Implementation and funding

The author(s) declare that no financial support was received for the research, authorship, and/or publication of this article.

The network's methodology will enable a comprehensive evaluation of innovative techniques and the establishment of a multistakeholder task force, including patient advocates and policymakers, to implement the most efficient strategies into the public health system across all regions, with the aim of promoting equity in achieving the objective to “cure more and cure better”.

At present, the ReALLNet infrastructure has been funded through the support of pharmaceutical companies and the support of SEHOP.

The SEHOP-PENCIL study (PMP21/00073 AES-ISCIII), within its aim of implementing personalized medicine programme for pediatric oncology in Spain, has identified ReALLNet as an efficient platform focused and high specialized on malignant hematological diseases. As a result, it has established common working groups between both projects, and provide financial support for part of oncogenomic studies offered by ReALLNet.

We also plan to finance research modules through competitive grant.

## 9. Conclusion

Research focused on “what's next” in the diagnosis and management of pediatric R/R ALL patients offers significant opportunities to improve responses and reduce toxicity through more tailored treatments. To integrate this research into bedside patient care in a pragmatic and real-life context, it is crucial to organize this innovation in a comprehensive and accessible manner for all treating physicians.

ReALLNet is an interdisciplinary and multicentric network within the Spanish scientific academic community of SEHOP. It establishes a platform to introduce and validate both existing and emerging diagnostic techniques, alongside innovative research, in a collaborative environment that connects experts in diagnosis, management, research, and computation in R/R ALL to every pediatric oncology department in Spain. Its primary objective is to equip all physicians with the latest diagnostic and decision-making resources, while also providing a systematically organized data registry and sample biobank to advance research in pediatric R/R ALL.

## Data Availability

The original contributions presented in the study are included in the article/Supplementary Materials, further inquiries can be directed to the corresponding authors.
